# The microbiome in mind: Reflections on antibiotic prescription in primary care

**DOI:** 10.4102/safp.v67i1.6238

**Published:** 2025-12-19

**Authors:** Nicolas D B. Roos

**Affiliations:** 1 Department of Pathology, Faculty of Health Sciences, University of the Witwatersrand, Johannesburg, South Africa

**Keywords:** antimicrobial stewardship, infectious diseases, microbiome, paediatrics, primary care

## Abstract

In this opinion piece, the author critically reflects on his own antimicrobial stewardship practices in primary care and the complex factors influencing antibiotic prescription, specifically in children.

**Contribution:**

In light of emerging evidence of the role of the microbiome in both health and disease, this piece raises a key question: Are we doing more harm than good?

## Introduction

‘Doctor, I know my body. If I don’t get antibiotics, I won’t get better’. This comment, which I have heard countless times in primary care, reflects a deeply ingrained belief that recovery is dependent on antibiotics. Having worked in an urban general practice in Cape Town’s northern suburbs for the past year, I have experienced first-hand the pressure to prescribe antibiotics and the poor stewardship that goes along with it. I am writing this opinion piece with self-conscious doubt about my own prescribing practices.

The role and make-up of our microbiomes are incredibly complex topics. As little as we know about deep space or the ocean floor, we likely know just as little about our own endogenous microbial communities, an integral part of our wellness.^1,2^

## Personal experience

In my experience, the idea that antibiotics are medicines used to treat ‘colds and flus’ has become entrenched in public perception to the extent that this prescription is being expected. A few months into working as a general practitioner, I encountered a 2-year-old boy with viral nasopharyngitis. The patient’s caregiver was upfront about requesting antibiotics, which I advised against. This developed into an argument, which finally led to me conceding, only to later discover that in his two-and-a-half years of life, he had been on 16 courses of antibiotics. I started having discussions with parents and realised that this is not an isolated incident, albeit a severe one, and that children typically receive multiple antibiotic courses per year. As it stands, South Africa does not monitor antimicrobial usage in the private primary care sector.^3^ In the private sector, antimicrobial usage is only monitored at the hospital level; therefore, we have limited data as to the extent of this problem in private primary care. It is important to note that globally, in both high- and low- to middle-income countries, primary care is the largest contributor to antibiotic prescriptions.^4,5^ In my personal experience, the issue of unnecessary antibiotic prescriptions in private primary care is pervasive and severe.

Only recently have we begun to understand the role that our microbiomes play in our health and in pathology. We are beginning to understand the significant impact of dysbiosis, with it being linked to atopy,^6^ obesity, diabetes and cardiovascular pathology,^7^ among others. Yet, with mounting evidence, we continue to prescribe antibiotics without indication. I started to ask myself why. Why are antibiotics used so liberally? Why do we feel so dependent on them? Is pressure from concerned parents to blame, or are we as medical practitioners more at fault? Is knowledge about antimicrobial stewardship lacking, or do we know what is right yet alter our behaviour to avoid upsetting patients and losing clients?

Having been a young general practitioner needing to build a patient base, I soon realised the importance of keeping patients happy. This necessity to satisfy patients often opposed good clinical practice, never more so than with antibiotic prescriptions. I felt compelled to comply with patient demands to ensure my financial stability, and my own personal interest became a factor in how I treated my patients. This avoidance of tension in the consultation, and the need to balance patient expectation with good clinical practice, has been identified in primary care centres globally.^8^ It has been well established that patient expectations influence general practitioners’ decision to prescribe antibiotics.^9^ I noticed myself becoming more liberal with antibiotic scripts to the point where I would prescribe them without clear indication and even without parents asking for it, just to avoid an argument. In 2020, Mathibe et al. found that 76% of children with acute upper respiratory tract infections in Pietermaritzburg were prescribed antibiotics while only 33% of parents specifically asked for it.^10^ Are we overcompensating to avoid these conversations with patients?

The old adage goes: ‘Treat a cold, it lasts for a week. Don’t, and it lasts for seven days’. I often advise parents of children with viral nasopharyngitis not to treat it. I simply recommend using paracetamol in the case of fevers, but that no other medications or antibiotics are needed. More often than not, this is received negatively. Have we, as a society, stopped normalising these viral illnesses? Understandably, both parents and physicians want to feel in control and able to help. Unfortunately, we have very little to no evidence for most symptomatic treatments for the common cold, especially in children under 4 years of age.^11^ This is supported by regulatory authorities such as the US Food and Drug Administration (FDA) recommending against the use of over-the-counter cough and cold medicine in children less than 4 years of age^12^ and South African Health Products Regulatory Authority (SAPHRA) in children less than 2-years-old.^13^ This is a vulnerable age group, and we as clinicians can often feel compelled to offer some sort of treatment rather than just watching and waiting, leading to the prescription of antibiotics. However, this is also an age group with an immature gut microbiome susceptible to the effects of antibiotics and the sequelae of dysbiosis.^14^

## Evidence and studies

Having initially entered university as a nature conservation student, I have found striking similarities between our declining ecosystems and the disruption of our own microbiomes. Our ‘macrobiomes’, so to speak, such as coral reefs and rainforests, flourish in diversity. When a species is lost, we find chain events leading to further disruptions negatively impacting other species and the ecosystem as a whole. With more than a thousand microorganism species already found to form part of our gut, and with this estimated to only be a fraction of all species involved,^15^ our gut is proving no different from any other biome on earth, flourishing in diversity. Studies indicate a decreased level of microbiome diversity in various autoimmune and cardiometabolic pathologies.^16,17,18^ Even in environmental organisms, microbial diversity has been found to have an inverse relationship with the prevalence of antimicrobial resistance genes.^19^ In contrast, studies indicate that antibiotic exposure causes a prolonged decrease in gut microbiome alpha diversity for up to 2 years.^20^ Considering children are receiving multiple antibiotic courses per year, this is cause for concern.

In an attempt to improve antimicrobial stewardship, I focused on educating my patients, using statistics to strengthen my argument. While it is estimated that, in children, only 8% of upper respiratory tract infections complicate with secondary bacterial infection,^21^ these infections are regularly cited as one of the most common reasons for antibiotic prescriptions. In addition, antibiotics are mostly prescribed empirically in these cases, with no bacteriological evidence to support treatment.^22^ I have found educating my patients and shared decision-making to be useful tools, rather than simply refusing antibiotic prescriptions. In addition, multiple other methods have been shown to improve antibiotic prescriptions. These include point-of-care testing like Streptococcal antigen testing, the use of clinical decision support tools and prescribing antibiotics as back-up and educating the patient on when to use it.^23^ In the South African context specifically, audit and feedback meetings significantly improved adherence to guidelines.^24^

## Strengthening stewardship

When facing a paediatric patient with an upper respiratory tract infection, both local and international guidelines advise to focus on certain symptoms that point to a viral versus a bacterial cause. Bacterial sinusitis should be considered only when the symptoms are persistent and not improving for 10 days (or 3–4 days if severe) or when double-sickening occurs.^26^ With pharyngitis or tonsilitis, a bacterial cause should be considered in the presence of tender anterior cervical lymphadenopathy, tonsillar exudates and significant fevers (> 38°C).^27^ Rhinorrhoea, cough and conjunctivitis often point to a viral cause.

Various antimicrobial stewardship courses are available to support clinicians with ongoing professional development. For those aiming to strengthen their prescribing practices, consider the following free courses:

- Antimicrobial Stewardship: Improving Clinical Outcomes by Optimization of Antibiotic Practices, by Stanford University (https://online.stanford.edu/courses/som-ycme0001-antimicrobial-stewardship-improving-clinical-outcomes-optimization-antibiotic)
- Antimicrobial Stewardship: A Competency-Based Approach, by the World Health Organization (https://whoacademy.org/coursewares/course-v1:WHOAcademy-Hosted+H0098EN+H0098EN_Q4_2024?source=edX)

## Conclusion

In 1860, the Boston physician Jacob Bigelow published his Brief Expositions of Rational Medicine. In it, he expressed his disillusionment with the prevailing ‘heroic’ medical practices of the time, practices with little evidence, often resulting in harm and famously observed: ‘… the amount of death and disaster in the world would be less, if all disease were left to itself …’.^25^ Every doctor in this country pledges themselves to the Hippocratic Oath, the key principle being ‘primum non nocere’ – first, do no harm. Are we doing more harm than good to these children? Would a child with a common cold not have been better off not visiting their doctor at all, waiting for the symptoms to pass, rather than receiving an antibiotic? Would their microbiomes have had a better chance of maturing, reducing their risk for atopy, obesity and the growing list of pathologies linked to dysbiosis? Do Jacob Bigelow’s words still hold some truth today? This article is not meant to judge; I have been in the same position of poor antimicrobial stewardship practices and continuously ask myself whether I have harmed my paediatric patients. There are multiple facets to this complex issue, from financial incentives in keeping patients satisfied to doubt in our ability to discern viral from bacterial pathologies in a susceptible population such as young children. However, we do owe it to our children to critically analyse our own prescribing practices without bias and, above all, to first do no harm.
